# Supplementary material for: Surveillance of Infections and Antibiotic Use in 21 Nursing Home Wards during the COVID-19 Pandemic: A Systematic Assessment
Source: Int J Environ Res Public Health. 2024 Mar 18;21(3):358. doi: 10.3390/ijerph21030358 (PMC10970298; doi:10.3390/ijerph21030358)
Supplement: Supplementary file 1 [file ijerph-21-00358-s001.zip › ijerph-2912240-supplementary.pdf]

Report questionnaire:

Code number:

Date:

How many patients have an infection?

- 0
- 1
- 2
- 3
- 4
- 5
- 6
- 7
- 8
- 9
- 10

Numbers of residents transferred to hospital due to suspected infection the last week:

- None
- 1
- 2
- 3
- 4
- Over 5

Numbers of deaths the last week, with infection was listed as primary or secondary diagnosis?

- None
- 1
- 2
- 3
- 4
- Over 5

For each patient referenced in question 1, all of the following questions would be posed:

Patient 1:

Gender:

- Female
- Male

Age:

- Under 85 years old
- 85 years old or above

The infection has…

- Arisen in the last week
- Continued for over a week

Type of infection:

- Respiratory tract infection
- Skin-tissue infection
- Urinary tract infection
- Sepsis
- Gastrointestinal infection
- Other

If other, what type of infection:

If it's a urinary tract infection, does the resident have a permanent urinary catheter?

- Yes
- No

Does the resident get antibiotic treatment?

- Yes
- No

If yes, type of antibiotic:

- Amoxicillin
- Trimethoprim-sulfamethoxazole
- Pivmecillinam
- Dicloxacillin
- Ciprofloxacin
- Phenoxymethyl-penicillin
- Trimethoprim
- Cefotaxime
- Combination/multiple types
- Metronidazole
- Other

If other, what type of antibiotic:

Method of administration:

- Per os
- Intravenous

Date when antibiotic treatment was initiated

Expected number of treatment days:

Does the resident have multiple infections?

- Yes
- No

If yes, all questions under patient 1, excluding those about gender and age, would be repeated.

Other comments?

Questionnaire about sociodemographic variables:

Code number:

Type of ward:

- Long-term ward
- Short-term ward
- Other

If other, what type of ward:

Numbers of beds in the ward:

How many residents does the ward have at the moment?

How many of the residents are women?

How many of the residents are 85 years old or older?

How many job positions does the ward have?

How many of these are nurses’ positions?

Other comments?
